# Omicron variant and pulmonary involvements: a chest imaging analysis in asymptomatic and mild COVID-19

**DOI:** 10.3389/fpubh.2024.1325474

**Published:** 2024-07-04

**Authors:** Peiben Liu, Kejun Cao, Guanqun Dai, Tingzhen Chen, Yifan Zhao, Hai Xu, Xiaoquan Xu, Quan Cao, Yiyang Zhan, Xiangrong Zuo

**Affiliations:** ^1^Department of Critical Care Medicine, The Second Hospital of Nanjing, Affiliated to Nanjing University of Chinese Medicine, Nanjing, Jiangsu, China; ^2^Department of Critical Care Medicine, The First Affiliated Hospital of Nanjing Medical University, Nanjing, Jiangsu, China; ^3^Department of Comprehensive Internal Medicine, The First Affiliated Hospital of Nanjing Medical University, Nanjing, Jiangsu, China; ^4^Department of Radiology, The First Affiliated Hospital of Nanjing Medical University, Nanjing, Jiangsu, China

**Keywords:** chest imaging, clinical features, lung total severity score, pulmonary involvements, SARS-CoV-2 omicron variant

## Abstract

**Objectives:**

To identify clinical characteristics and risk factors for pulmonary involvements in asymptomatic and mildly symptomatic patients infected with SARS-CoV-2 Omicron variant by chest imaging analysis.

**Methods:**

Detailed data and chest computed tomography (CT) imaging features were retrospectively analyzed from asymptomatic and mildly symptomatic patients infected with Omicron between 24 April and 10 May 2022. We scored chest CT imaging features and categorized the patients into obvious pulmonary involvements (OPI) (score > 2) and not obvious pulmonary involvements (NOPI) (score ≤ 2) groups based on the median score. The risk factors for OPI were identified with analysis results visualized by nomogram.

**Results:**

In total, 339 patients were included (145 were male and 194 were female), and the most frequent clinical symptoms were cough (75.5%); chest CT imaging features were mostly linear opacities (42.8%). Pulmonary involvements were more likely to be found in the left lower lung lobe, with a significant difference in the lung total severity score of the individual lung lobes (*p* < 0.001). Logistic regression analysis revealed age stratification [odds ratio (OR) = 1.92, 95% confidence interval (CI) (1.548–2.383); *p* < 0.001], prolonged nucleic acid negative conversion time (NCT) (NCT > 8d) [OR = 1.842, 95% CI (1.104–3.073); *p* = 0.019], and pulmonary diseases [OR = 4.698, 95% CI (1.159–19.048); *p* = 0.03] as independent OPI risk factors.

**Conclusion:**

Asymptomatic and mildly symptomatic patients infected with Omicron had pulmonary involvements which were not uncommon. Potential risk factors for age stratification, prolonged NCT, and pulmonary diseases can help clinicians to identify OPI in asymptomatic and mildly symptomatic patients infected with Omicron.

## Introduction

Four years have passed since the outbreak of the coronavirus disease (COVID-19) pandemic began, and as of 17 Mar 2024, there have been 7,040,264 deaths reported to the World Health Organization (WHO) (1). The impact of COVID-19 has been profound, affecting the global economy, environment, and tourism, among other sectors. Between 2019 and 2020, the number of unemployed people worldwide increased from 191.93 million to 235.21 million, highlighting just a fraction of the pandemic’s socioeconomic ramifications (2). Consequently, countries worldwide have undertaken extensive efforts to curb the spread of the virus, including vaccine development, reinforced prevention and control measures, and implementation of supportive policies (3, 4). However, as a result of genetic variations in SARS-CoV-2 during viral replication to evade the human immune system and achieve self-protection, several SARS-CoV-2 variants have been reported worldwide (5, 6), with the Omicron variant notably becoming predominant (7, 8). Although the Omicron variant has been proven to spread rapidly with less virulence, causing mainly asymptomatic and mildly symptomatic infections which tends to the upper respiratory tract, not the lungs (7, 9), the research has revealed that even among these asymptomatic and mildly symptomatic patients with Omicron who received limited medical attention, some experienced severe outcomes, particularly among the older adult (10). This prompts the hypothesis that potential pulmonary involvements may occur in asymptomatic and mildly symptomatic patients with Omicron, underscoring the importance of early identification to mitigate disease progression.

Chest computed tomography (CT) has become a standard tool for clinicians to evaluate the extent of pulmonary involvement in patients with COVID-19 (11). The Radiological Society of North America (RSNA) guidelines have categorized chest images of COVID-19 pneumonia into four classifications to discern the correlation between pulmonary involvements and COVID-19 (12). While several studies have devised scoring systems to quantify the severity of pulmonary involvements in COVID-19 pneumonia based on chest CT images (13–19), they have predominantly focused on symptomatic patients, neglecting asymptomatic and mildly symptomatic cases. Consequently, there is a paucity of research regarding the chest imaging characteristics of pulmonary involvements in asymptomatic and mildly symptomatic patients with Omicron. Thus, the objective of our study is to elucidate chest CT imaging features indicative of pulmonary involvements in asymptomatic and mildly symptomatic patients with Omicron and to develop a visual model for identifying individuals with potential pulmonary involvements warranting further evaluation via chest CT scans.

## Materials and methods

### Patients and clinical data

Our study was retrospective in nature and approved by the Ethics Committee (Approval No. 2022-SR-491). All complete data were retrospectively collected from cases infected with Omicron admitted to Shanghai Lin-gang Shelter Hospital from 24 April to 10 May 2022, and the data were de-identified and anonymously analyzed. Population screening was performed according to the following inclusion criteria: (1) patients with the Omicron variant diagnosed in accordance with the Chinese COVID-19 treatment guidelines with asymptomatic and mildly symptomatic disease (20). All patients tested positive for the SARS-CoV-2 nucleocapsid protein gene (*N* gene)/open reading frame 1ab gene (*ORF1ab* gene) using real-time fluorescent quantitative PCR (RT-qPCR) before admission; (2) age ≥ 18 years; (3) finger pulse oximetry >94%; (4) no severe organ dysfunction; (5) asymptomatic patients with clinical symptoms and mildly symptomatic patients with severe or prolonged respiratory symptoms after admission; and (6) chest CT examination was determined necessary by doctors. The exclusion criteria were (1) age < 18 years; (2) finger pulse oximetry ≤93%; (3) respiratory rate ≥ 30 breaths/min; (4) heart, lung, renal, and other important organ dysfunctions; (5) uncontrolled underlying diseases such as hypertension, diabetes, coronary artery disease, and psychiatric diseases; (6) inability to follow up the nucleic acid test results because of serious illness or the need for special treatment (such as chemotherapy); and (7) serious clinical data deficiencies. The data obtained included age, sex, stage, history of vaccination, comorbidities, and major clinical symptoms (e.g., respiratory, gastrointestinal, and other systemic symptoms).

### Nucleic acid detection methods and negative conversion time

Specimens were collected from the nasopharynx and oropharynx, and the first test was performed within 24 h after the patients were admitted to the shelter hospital. Subsequently, swab specimens were collected once a day at an interval of 24 h and tested by Shanghai Dean Medical Laboratory Co. Nucleic acid test negativity was judged by two consecutive SARS-CoV-2 nucleic acid tests with Ct values ≥35 for both the *N* and *ORF1ab* gene (RT-qPCR with a cut-off value of 40 and a sampling interval of at least 24 h). The NCT was the time from the date of the first positive nucleic acid test to the date of specimen collection of the first negative nucleic acid test (two consecutive negative tests).

### Interpretation of omicron infection

During our study period, Omicron was the predominant strain of COVID-19, and all newly identified viral genomes in Shanghai belonged to the BA.2.2 sub-lineage of the Omicron variant of SARS-CoV-2 (B.1.1.529) (10, 21). Consequently, patients in our study were considered infected with Omicron, and molecular analyses for Omicron identification were not conducted.

### Acquisition and interpretation of chest CT images

The patient’s first chest CT images were analyzed during hospitalization. Patients were scanned in the supine position with a continuous spiral scan from the lung apex to the lung base. Two radiologists with more than 10 years of experience in the cardiothoracic field reviewed the images individually, and in case of disagreement, another radiologist with more than 15 years of experience made a comprehensive judgment. The total severity score system was selected for the visual evaluation of pulmonary involvements on chest CT, and TSS was checked and counted by a statistician. The TSS system was based on previously published methods (22).

### Statistical analysis

Excel 2016 (Microsoft Corp., Redmond, WA) was used to collect the data. Continuous variables were compared using the Mann–Whitney U test. Categorical variables were compared using the χ^2^-test, Fisher’s exact test or Yates’ correction. The variables of multiple groups were compared using a one-way analysis of variance (ANOVA). The binary logistic regression was used to identify the factors associated with OPI and variables assessed *p* < 0.1 by univariate analysis could be incorporated into multivariate analysis. Statistical analyses were conducted using SPSS version 25.0 (IBM Corp., Armonk, NY) and R software (version 4.2.2^1^; R Foundation for Statistical Computing, Vienna, Austria). The reported statistical significance levels were two-sided, with statistical significance set at *p* < 0.05.

## Results

### Basic information

**Figure 1:**
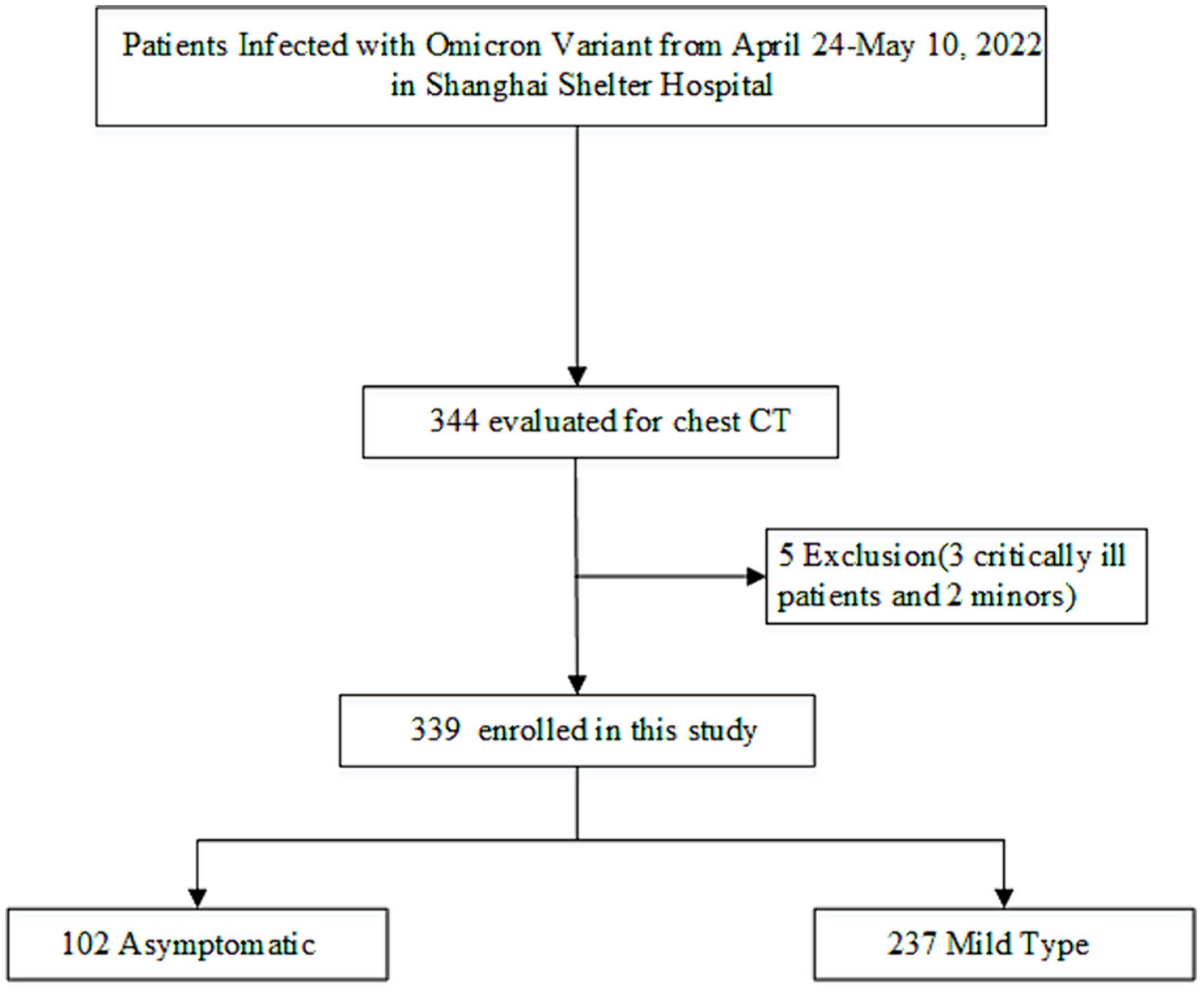
Flowchart of study patients.

In accordance with the inclusion criteria, 344 patients were screened; three seriously ill patients and two minors were excluded according to the exclusion criteria. Finally, 339 patients were included in the study (Figure 1). The median age of all patients was 57 years [interquartile range (IQR), 44–69 years], and 145 patients (42.8%) were male; 257 patients (24.4%) were aged ≥70 years, and 155 patients (45.7%) had underlying diseases. In addition, 112 patients (33.0%) were unvaccinated, and the median NCT was 8 days (IQR, 5–10 days).

The baseline clinical characteristics of the asymptomatic and mildly symptomatic patients are shown in Table 1. The differences were mainly in clinical symptoms, with significant differences in cough (80.6%), sputum (68.4%), stuffy nose (15.2%), muscle aches (22.4%), and malaise (28.3%) in mildly symptomatic patients compared with asymptomatic patients. Notably, no difference was found in vaccination status and NCT between the asymptomatic and mildly symptomatic groups, although the mildly symptomatic group included patients with a longer NCT.

**Table 1:** Baseline clinical characteristics, clinical symptoms, vaccination status, and NCT of the study patients.

Characteristic	Asymptomatic patients (*n* = 102)	Mild type patients (*n* = 237)	*p*-value
Age (years)	58 (46–68)	56 (43–69)	0.726^c^
<70 years	79 (77.5)	178 (75.1)	0.746^a^
Gender			0.835^a^
Female	45 (44.1)	100 (42.2)	
Male	57 (55.9)	137 (57.8)	
Chronic diseases	41 (40.2)	101 (42.6)	0.769^a^
Hypertension	29 (28.4)	79 (33.3)	0.446^a^
Diabetes	19 (18.6)	27 (11.4)	0.107^a^
Coronary heart disease	5 (4.9)	20 (8.4)	0.360^a^
Pulmonary diseases	3 (2.9)	12 (5.1)	0.566^b^
Rheumatic diseases	1 (1.0)	3 (1.3)	1^b^
Other diseases	13 (12.7)	42 (17.7)	0.327^a^
Clinical symptoms			
Fever	15 (14.7)	57 (24.1)	0.074^a^
Sore throat	21 (20.6)	60 (25.3)	0.425^a^
Cough	65 (63.7)	191 (80.6)	0.002^a^
Sputum	56 (54.9)	162 (68.4)	0.025^a^
Nose runny	9 (8.8)	26 (11.0)	0.688^a^
Nose stuffy	6 (5.9)	36 (15.2)	0.027^a^
Chills	5 (4.9)	21 (8.9)	0.301^a^
Muscle soreness	6 (5.9)	53 (22.4)	<0.001^a^
Fatigue	15 (14.7)	67 (28.3)	0.011^a^
Dyspnea	8 (7.8)	27 (11.4)	0.429^a^
Diarrhea	5 (4.9)	23 (9.7)	0.208 ^a^
Other symptoms	10 (9.8)	27 (11.4)	0.810^a^
Vaccination status			0.814^a^
Unvaccinated	35 (34.3)	77 (32.5)	
Single or double vaccinated	32 (31.4)	70 (29.5)	
Booster vaccinated	35 (34.3)	90 (38.0)	
Nucleic acid convention time (days)	7 (4–10)	8 (6–10)	0.162^c^
<8 days	46 (45.1)	84 (35.4)	0.120^a^

Data are presented as the median (interquartile range) or number (percentage) of patients. ^a^Determined with χ^2^-test. ^b^Determined with Fisher’s exact test. ^c^Determined with Mann–Whitney U test.

### Description of CT imaging features

CT imaging features of 339 patients were analyzed according to the type of parenchymal opacity, opacity (axial) distribution, airway changes, underlying pulmonary changes, and other involvements on CT.

Regarding the type of parenchymal opacity, 16 patients (4.7%) had consolidation, with 71 cases (20.9%) of ground glass opacity (GGO); 145 patients (42.8%) had linear opacities, rounded morphology was found in 56 patients (16.5%), and 123 patients (36.3%) had nodules. Interestingly, four patients (1.2%) had reverse halo signs, and only one (0.3%) had a crazy paving pattern. Whereas the opacities were mainly distributed peripherally (44.8%), only four patients (1.2%) showed a central distribution (peribronchovascular), and the remaining 98 patients (28.9%) showed no axial lung distribution. In the study cohort, airway changes were not significantly manifested; in particular, bronchial wall thickening was observed in five cases (1.5%), bronchiectasis in 17 cases (5.0%), and none of the patients had airway secretions. Fifty-five patients (16.2%) showed signs of pulmonary emphysema, whereas pulmonary fibrosis was present in 17 patients (5.0%). The number of patients with pleural effusion and lymphadenopathy was 9 (2.7%) and 21 (6.2%), respectively, while none of the patients had hollow nodules. Finally, other involvements included pulmonary texture thickening and calcification, which were present in 59 cases (17.4%). The CT imaging features of the asymptomatic and mildly symptomatic groups are shown in Table 2.

**Table 2:** The type of parenchymal opacity, opacities (axial) distribution, airways, underlying lung lesions, and other findings of chest CT in two groups.

Findings	Asymptomatic patients (*n* = 102)	Mild type patients (*n* = 237)	*P*-value
Type of parenchymal opacity			
Consolidation (%)	4 (3.9)	12 (5.1)	0.785^b^
Ground glass opacities	23 (22.5)	48 (20.3)	0.740^a^
Linear opacities (%)	52 (51.0)	93 (39.2)	0.060^a^
Rounded morphology (%)	16 (15.7)	40 (16.9)	0.911^a^
Nodules (%)	37 (36.3)	86 (36.3)	1^a^
Reverse halo sign (%)	1 (1.0)	3 (1.3)	1^b^
Crazy-paving pattern	0 (0.0)	1 (0.4)	1^b^
Opacities (axial) distribution			
No axial lung distribution	30 (29.4)	68 (28.7)	0.997^a^
Central distribution (peribronchovascular)	0 (0.0)	4 (1.7)	0.32b^a^
Peripheral distribution	48 (47.1)	104 (43.9)	0.674^a^
Airways			
Bronchial wall thickening	1 (1.0)	4 (1.7)	1^b^
Bronchiectasi	3 (2.9)	14 (5.9)	0.381^a^
Airways secretions	0 (0.0)	0 (0.0)	
Underlying lung lesions			
Pulmonary emphysema	17 (16.7)	38 (16.0)	1^a^
Pulmonary fibrosis	2 (2.0)	15 (6.3)	0.156^a^
Other findings			
Pleural effusion	2 (2.0)	7 (3.0)	0.729^b^
Lymphadenopathy	8 (7.8)	13 (5.5)	0.562^a^
Pericardial effusion	2 (2.0)	2 (0.8)	0.587^b^
Hollow	0 (0.0)	0 (0.0)	
Others	14 (13.7)	45 (19.0)	0.310^a^

Data are presented as the number (percentage) of patients. ^a^Determined with χ^2^-test. ^b^Determined with Fisher’s exact test.

### The total severity score

**Figure 2:**
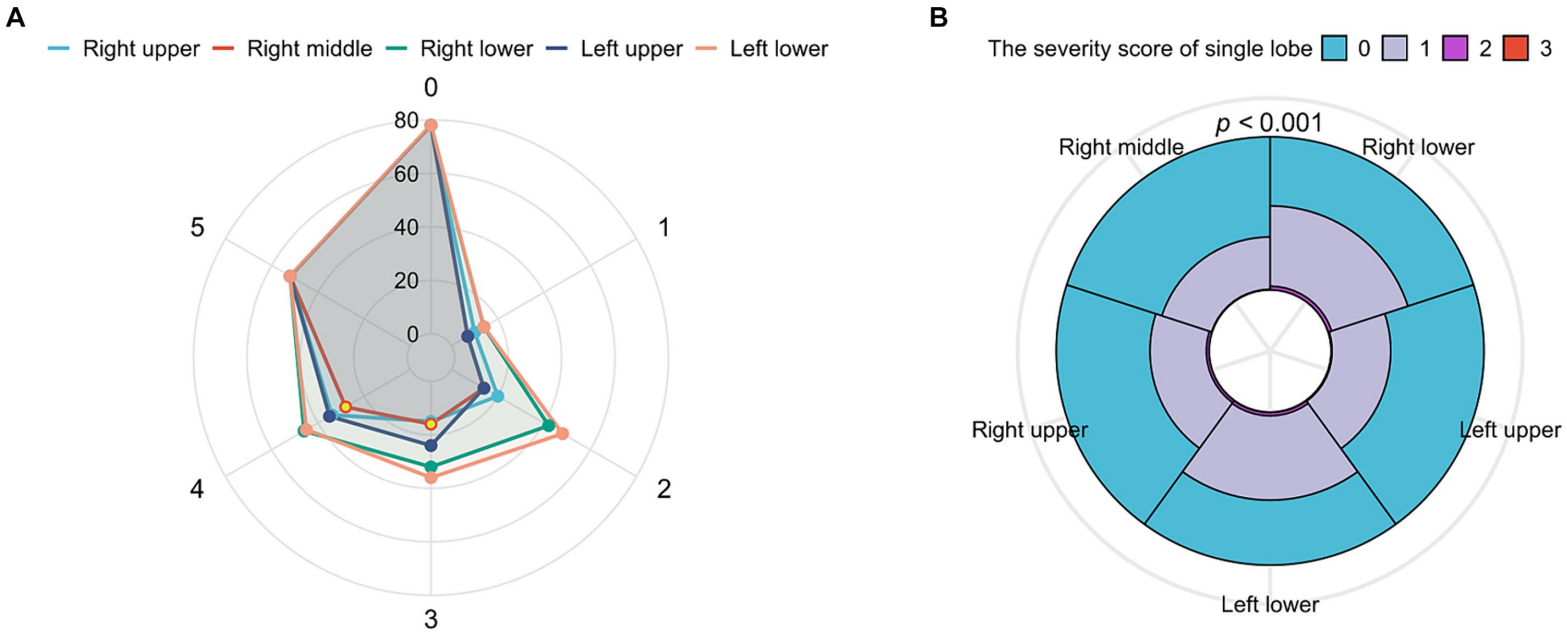
The analysis of total severity score (TSS) with lung lobes. **(A)** Radar chart depicting the characteristics of involved lobes. X axis shows the number of involved lobes (0–5), and y axis shows the counterpart number of patients. The wrap-around area of the left lobe was the largest, which represented that the left lobe was seriously involved, and the right lobe conversely. **(B)** Overlay of histograms showing the TSS’s characteristics of each lobe. The different color’s wrap-around area represented the counterpart number of patients. The cleaned data were counted accordingly to get the percentage of each subgroup in each category and determined with Yates’ correction. Radar chart and overlay of histograms were drawn for the data using the ggplot2 package.

The TSS was counted for the two groups, and the scores of all patients ranged from 0 to 10 with a median of 2 (IQR, 1–4). On the initial CT, all five lobes were involved in 52 patients (15.3%), four lobes in 47 (13.9%), three lobes in 41 (12.1%), two lobes in 69 (20.4%), and one lobe in 52 patients (15.3%), while only 78 patients (23.0%) had no pulmonary involvement. As shown in Figure 2A, the most frequently affected lobe was the left lower lobe (55.2%), with the least involvement in the right middle lobe (34.5%), and pulmonary involvement was most likely to be found in the left lower lobe, followed by the right lower lobe, left upper lobe, right upper lobe, and right middle lobe among patients with involvements in one to four lobes. The analysis of the severity of different lobe involvements revealed that only the left lower lobe had a score of 0–3, with the score ranging from 0 to 2 in all the other lobes. The data are shown in Figure 2B. The difference was statistically significant (*p* < 0.001), and we compared the TSS of the different lung lobes. Supplementary Table S1 shows the CT imaging features of the patients in the asymptomatic and mildly symptomatic groups, and there were no significant differences.

### The risk factors for obvious pulmonary involvements and developing the nomogram

**Figure 3:**
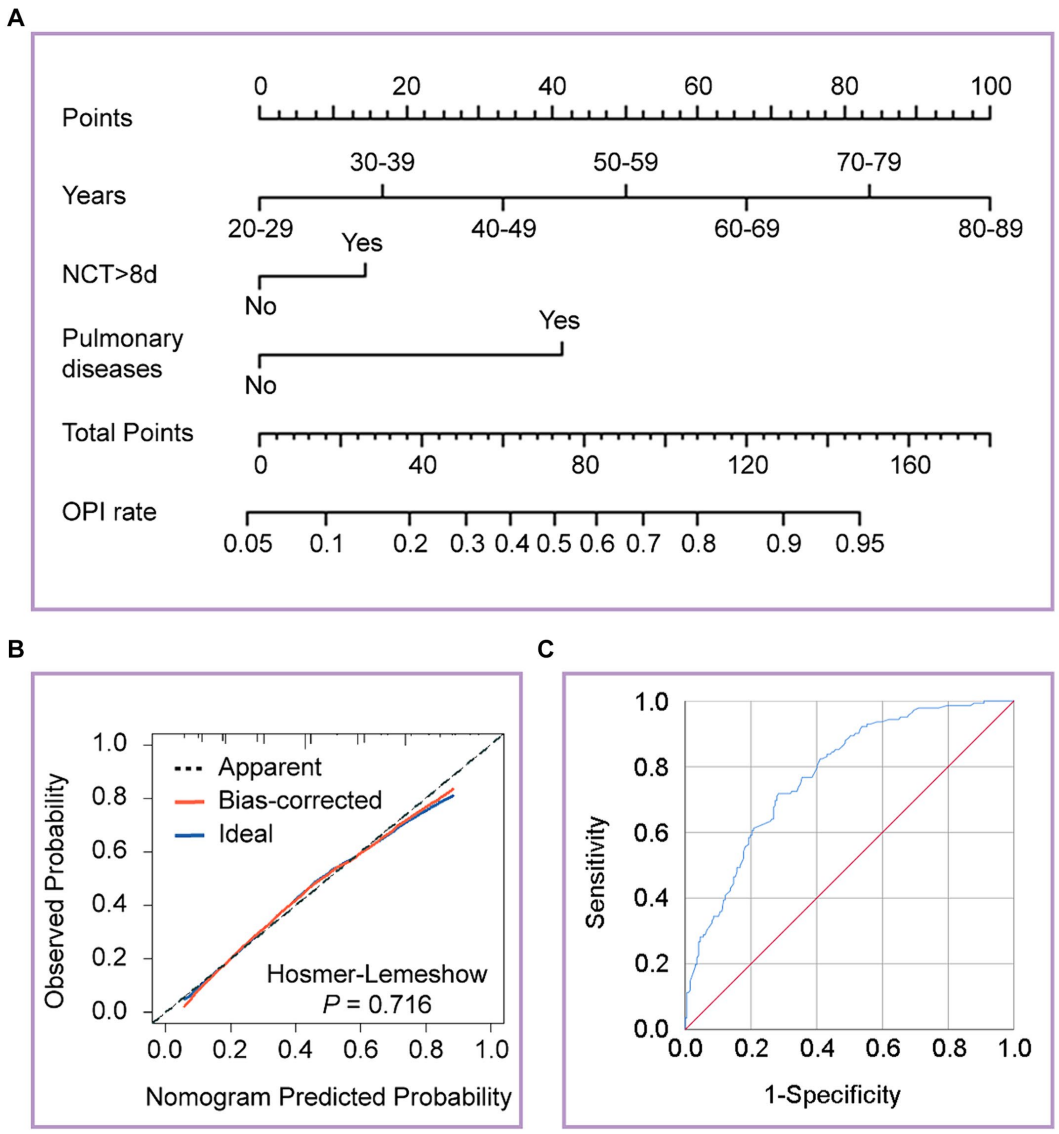
Developed the nomogram, calibration curves and receiver operating characteristic (ROC) curves of the nomogram. **(A)** The nomogram was developed on the regression model, with age stratification, prolonged NCT, and pulmonary diseases. **(B)** Calibration curves depict the calibration between the predicted and observed probability of OPI. The y-axis represents the observed OPI rate. The x-axis represents the predicted OPI risk. The diagonal dotted line represents a perfect prediction by an ideal model. The blue solid line represents the performance of the nomogram, of which a closer fit to the diagonal dotted line represents a better prediction. **(C)** ROC curves depict the diagnostic efficacy of the model. The y-axis represents the sensitivity of the model, and the x-axis represents specificity. The blue solid line represents a prediction by the nomogram.

We categorized the patients into OPI (TSS >2) and not OPI (NOPI) (TSS ≤2) groups based on the median TSS. The univariate binary logistic regression analysis of patients in both groups was shown in Table 3. Further multivariate analysis revealed age stratification [OR = 1.92, 95% CI (1.548–2.383); *p* < 0.001], prolonged NCT [OR = 1.842, 95% CI (1.104–3.073); *p* = 0.019], and pulmonary diseases [OR = 4.698, 95% CI (1.159–19.048); *p* = 0.03] were independent risk factors for the OPI group patients. To visually depict the effect of the age stratification, prolonged NCT and pulmonary diseases on the probability of OPI, we visualized the logistic regression model and plotted nomogram. As shown in Figure 3A, the total points obtained by summing the points corresponding to individual factors corresponded to the OPI rate with the vertical line of its score scale. Further, we verified the goodness of fit and detection efficacy of the nomogram using calibration curves and the receiver operating characteristic (ROC) curves (Figures 3B,C). The calibration curve illustrated that our constructed nomogram had a high goodness of fit (Hosmer–Lemeshow test, *p* = 0.716), while the value of the area under the ROC curve (AUC) of the model was 0.779.

**Table 3:** The univariate and multivariate binary logistic regression analysis used for identifying the risk factors for the obvious pulmonary involvements.

Variable	*P*-value	Variable	OR	95% CI	*P*-value
Age stratification	< 0.001	Age stratification	1.92	1.548–2.383	< 0.001
Vaccination status	0.002	NCT > 8d	1.842	1.104–3.073	0.019
NCT > 8d	0.015	Pulmonary diseases	4.698	1.159–19.048	0.03
Hypertension	0.067				
Coronary heart diseases	0.024				
Pulmonary diseases	0.006				
Other diseases	0.004				
Fever	0.099				
Muscle soreness	0.006				

## Discussion

In this study, we found that almost half of the asymptomatic and mildly symptomatic patients infected with Omicron had pulmonary involvements on CT, which were more likely to be found in the left lower lung lobe. Using the TSS system to assess the severity of pulmonary involvements, we revealed a significant difference in the TSS of the individual lung lobes (*p* < 0.001). Age stratification, prolonged NCT, and pulmonary diseases were identified as the risk factors for OPI by logistic regression analysis.

In our study, the proportion of asymptomatic infections was 30.1%, which is similar to the result of a previous meta-analysis study (32.40%) that included eight studies with 7,640 patients infected with the Omicron variant (23). Compared with the Delta variant (24), the prominent symptoms in patients with the Omicron variant were cough and sputum in our study, while upper respiratory tract symptoms (e.g., runny and stuffy nose) and systemic symptoms (e.g., fever, muscle soreness, and fatigue) occurred in less than 30% of patients. Geng et al. investigated the impact of symptoms on Omicron infection, identifying fever (OR = 6.358, 95% CI 1.748–23.119; *p* = 0.005) and diarrhea (OR = 6.523, 95% CI 1.061–40.110; *p* = 0.043) as risk factors for Omicron infection progression (10). However, logistic regression analysis in our study did not identify any clinical symptoms as risk factors for OPI, implying challenges in symptom-based identification of pulmonary involvements.

Multiple clinical and experimental studies have found that the virulence of the Omicron variant is significantly weaker than that of the wild-type strain and the Alpha, Beta, and Delta variants (25–28). The study found that Omicron infection has a lower CT severity score (CT-SS) than Delta infection (OR = −7.2, 95% CI (−9.9 to −4.5); *p* < 0.001) and the Delta variant has greater association with severe disease (OR = 4.6, 95% CI(1.2–26); *p* = 0.01) and admission to a critical care unit [OR = 7.0, 95% CI (1.5–66); *p* = 0.004] (28). Moreover, the lower virulence in the lungs, the main target of a viral attack, is reflected in pathological changes that are more symptomatic of acute exudation and less obvious changes in vascular damage and chronic fibrous exudation (29, 30). Compared with the wild-type strain and the Alpha, Beta, and Delta variants, patients with Omicron infection had nontypical peribronchovascular pneumonia and less pulmonary vascular involvement on chest CT images (28, 31). In our study, the types of parenchymal opacity were mainly linear opacities, nodules, GGO, and rounded morphology, whereas the reverse halo sign and crazy paving pattern were almost absent. Conversely, Uysal et al. observed that CT findings in asymptomatic COVID-19 patients primarily consisted of GGO, often localized peripherally (32). This discrepancy may be attributed to the variant types and suggests distinct areas of interest for different variants. Additionally, Tomris et al. (33) explored the distinct spatial distribution of angiotensin-converting enzyme 2 (ACE2) expression in Syrian hamster lung lobes infected with SARS-CoV-2, highlighting ACE2 predominance in the lower regions of the lung lobes, which likely contributes to pulmonary involvements primarily in these areas. Interestingly, the left lower lung lobe was most likely to be involved, while the right lower lung lobe was mostly involved in another study (34). The variations in spatial distribution of pulmonary involvement across different variants offer valuable insights into variant-specific pathogenic mechanisms.

Given the distinctive mutational profile of the virus, numerous radiographers and clinicians have endeavored to devise scoring systems aimed at evaluating the extent of pulmonary involvements on chest CT scans (13, 14). These systems serve the dual purpose of comprehending the impact of various mutant strains and guiding clinical decisions. The more established and widely recognized chest CT severity scoring systems are mainly the chest CT severity score (CT-SS) (15), chest CT score (16), TSS (17), modified TSS (18), and 3-level chest CT severity score (19). In a comparison of these scoring systems, Elmokadem et al. found that the TSS had a higher AUC (0.890) and shorter reporting time, whereas the chest CT score showed the highest specificity (95.2%) in discriminating severe cases (35). In our investigation, the TSS system was selected due to its inherent advantages, coupled with the requirement for detailed characterization of pulmonary involvement; the intricate lobe classification system of CT-SS risked undue dispersion of results, particularly as our study cohort comprised solely asymptomatic and mildly symptomatic cases. For the wild-type strain, one previous study reported a mean TSS of 9.9 (range, 0–19) (22), and another study showed a mean TSS of 9 (range, 4–12) (35). These studies further underscored a strong correlation between higher TSS and disease severity. Viceconte et al. compared TSS values between the Alpha and Delta variants, suggesting a higher TSS association with the Delta variant (36). Similarly, Inui et al. confirmed that patients with the Delta variant had a higher TSS (37) and that the Delta variant was more virulent (38), whereas patients with the Omicron variant exhibited a lower TSS. Our study revealed a median TSS of 2 (range, 0–10), with no significant variance observed between asymptomatic individuals and those presenting with mild symptoms.

We defined patients as having OPI when their TSS exceeded 2. The correlation between OPI and age in asymptomatic and mildly symptomatic patients with Omicron was not previously well known. Our study found age to be an independent risk factor for OPI, which might be due to the positive correlation between age and the number of copies of the virus (39), and another two studies proved that age was independent risk factors for the duration of viral shedding (40, 41). However, several studies (42, 43) did not reach this conclusion. Our study also observed that prolonged NCT indicated a higher level and longer duration of viral replication, with patients exhibiting prolonged NCT being more likely to develop OPI. While our study suggests that pulmonary disease may pose a risk factor for OPI, this conclusion necessitates further investigation, particularly given that patients with underlying pulmonary conditions may be more susceptible to Omicron infection. Other studies had found that the severity of pulmonary involvement was closely related to the variant (28, 31).

This study had several limitations and challenges. Firstly, the clinical symptoms of patients at initial admission to Shanghai Lin-gang Shelter Hospital were mainly collected using a WeChat Application, which may have led to data bias. Secondly, owing to the lack of nucleic acid detection and molecular analyses for Omicron identification in the early period, the time of the first positive nucleic acid test lagged behind the actual infection time in some patients, resulting in a shorter NCT than before, and the diagnosis of Omicron infection based on epidemiological inference might have bias in analysis of baseline data; then, only asymptomatic and mildly symptomatic patients infected with Omicron were analyzed and not compared with previous variants. In addition, Shanghai Lin-gang Shelter Hospital did not carry out laboratory tests in the early period owing to the lack of necessary conditions; hence, we could not provide the laboratory results of these patients. As a result of not reviewing the chest CT images and not following up, we lacked prognostic data, which limited the study to a description of the phenomena, and we could not analyze the clinical outcomes. It is necessary that clinicians should pay greater attention to the pathophysiological mechanisms and assess the follow-up chest CT findings to understand the spatio-temporal variations of the pulmonary involvements in mildly symptomatic and asymptomatic patients infected with Omicron, which will be of benefit in preventing disease progression.

## Conclusion

In conclusion, asymptomatic and mildly symptomatic patients infected with Omicron mainly had upper respiratory symptoms, with few and mild systemic symptoms, and Chest CT imaging features were dominated by linear opacities and GGO. However, pulmonary involvements on chest CT were not uncommon. Age stratification, prolonged NCT, and pulmonary diseases were risk factors for OPI, and we developed a nomogram to predict the risk for pulmonary involvements in asymptomatic and mildly symptomatic patients. We hope that our model can be effectively applied in a scenario where clinicians decide whether to have asymptomatic and mildly symptomatic patients finished CT, neither ignoring patients with underlying pulmonary involvements nor performing redundant CT scans.

## Data availability statement

The raw data supporting the conclusions of this article will be made available by the authors, without undue reservation.

## Ethics statement

The studies involving humans were approved by the Ethics Committee of the First Affiliated Hospital of Nanjing Medical University. The studies were conducted in accordance with the local legislation and institutional requirements. Written informed consent for participation was not required from the participants or the participants’ legal guardians/next of kin because our study was retrospective in nature.

## Author contributions

PL: Data curation, Formal analysis, Methodology, Visualization, Writing – original draft, Writing – review & editing. KC: Formal analysis, Investigation, Writing – review & editing. GD: Conceptualization, Data curation, Resources, Writing – review & editing. TC: Validation, Visualization, Writing – review & editing. YifZ: Validation, Visualization, Writing – review & editing. HX: Methodology, Software, Writing – review & editing. XX: Methodology, Software, Writing – review & editing. QC: Project administration, Supervision, Writing – review & editing. YiyZ: Project administration, Supervision, Writing – review & editing. XZ: Data curation, Funding acquisition, Resources, Writing – review & editing.

## Footnotes

^1^http://www.Rproject.org
