# Supplementary material for: Omicron variant and pulmonary involvements: a chest imaging analysis in asymptomatic and mild COVID-19
Source: Front Public Health. 2024 Jul 4;12:1325474. doi: 10.3389/fpubh.2024.1325474 (PMC11258674; doi:10.3389/fpubh.2024.1325474)
Supplement: Supplementary file 1 [file Table_1.DOCX]

**Table S1** Comparison of TSS of two groups

| Findings | Asymptomatic Patients(n=102) | Mild Type patients (n=237) | *P* Value |
| --- | --- | --- | --- |
| Lobe Involvement |  |  |  |
| Right upper lobe | 39 (38.2) | 92 (38.8) | 1^a^ |
| Right upper lobe score |  |  | 0.876^b^ |
| 0 | 63 (61.8) | 145 (61.2) |  |
| 1 | 38 (37.3) | 87 (36.7) |  |
| 2 | 1 (1.0) | 5 (2.1) |  |
| Right middle lobe | 37 (36.3) | 80 (33.8) | 0.747^a^ |
| Right middle lobe score |  |  | 0.734^b^ |
| 0 | 65 (63.7) | 157 (66.2) |  |
| 1 | 37 (36.3) | 79 (33.3) |  |
| 2 | 0 (0.0) | 1 (0.4) |  |
| Right lower lobe | 58 (56.9) | 128 (54.0) | 0.715^a^ |
| Right lower lobe score |  |  | 0.526^b^ |
| 0 | 44 (43.1) | 109 (46.0) |  |
| 1 | 57 (55.9) | 121 (51.1) |  |
| 2 | 1 (1.0) | 7 (3.0) |  |
| Left upper lobe | 42 (41.2) | 90 (38.0) | 0.665^a^ |
| Left upper lobe score |  |  | 0.657^b^ |
| 0 | 60 (58.8) | 147 (62.0) |  |
| 1 | 42 (41.2) | 88 (37.1) |  |
| 2 | 0 (0.0) | 2 (0.8) |  |
| Left lower lobe | 58 (56.9) | 137 (57.8) | 0.967^a^ |
| Left lower lobe score |  |  | 0.881^b^ |
| 0 | 44 (43.1) | 100 (42.2) |  |
| 1 | 57 (55.9) | 130 (54.9) |  |
| 2 | 1 (1.0) | 6 (2.5) |  |
| 3 | 0 (0.0) | 1 (0.4) |  |
| TSS | 2 (1-4) | 2 (1-4) | 0.810^c^ |

Data are presented as the median (interquartile range) or number (percentage) of patients.

^a^ Determined with χ^2^ test

^b^ Determined with Fisher’s exact test

^c^ Determined with Mann–Whitney U test

**Table S2** The significantly different characteristics of three groups categorized by vaccination status

| Characteristic | Unvaccinated (n=112) | Single or double vaccinated(n=102) | Booster vaccinated (n=125) | *P* Value ^a^ |
| --- | --- | --- | --- | --- |
| Male | 63 (56.2) | 49 (48.0) | 82 (65.6) | 0.028 |
| Age (years) | 68 (56-74) | 53 (37-61) | 53 (44-63) | <0.001 |
| <70 years | 65 (58.0) | 89 (87.3) | 103 (82.4) | <0.001 |
| Chronic Diseases | 67 (59.8) | 36 (35.3) | 39 (31.2) | <0.001 |
| Hypertension | 48 (42.9) | 30 (29.4) | 30 (24.0) | 0.006 |
| Diabetes | 23 (20.5) | 7 (6.9) | 16 (12.8) | 0.014 |
| Coronary heart disease | 15 (13.4) | 3 (2.9) | 7 (5.6) | 0.009 |
| Other Diseases | 32 (28.6) | 8 (7.8) | 15 (12.0) | <0.001 |
| Clinical Symptoms |  |  |  |  |
| Fever | 14 (12.5) | 33 (32.4) | 25 (20.0) | 0.002 |
| Cough | 80 (71.4) | 72 (70.6) | 104 (83.2) | 0.042 |
| Nose runny | 6 (5.4) | 9 (8.8) | 20 (16.0) | 0.023 |
| Nose stuffy | 5 (4.5) | 14 (13.7) | 23 (18.4) | 0.004 |
| Muscle soreness | 8 (7.1) | 22 (21.6) | 29 (23.2) | 0.002 |
| Fatigue | 14 (12.5) | 33 (32.4) | 35 (28.0) | 0.001 |
| TSS | 3 (1-5) | 2 (0-3) | 2 (1-4) | 0.001 |
| Ground glass opacities | 33 (29.5) | 19 (18.6) | 19 (15.2) | 0.021 |
| Central distribution (peribronchovascular) | 0 (0.0) | 0 (0.0) | 4 (3.2) | 0.037 |

Data are presented as the median (interquartile range) or number (percentage) of patients.

^a^ Determined with one-way ANOVA
